# Smartwatch Measures of Outdoor Exposure and Myopia in Children

**DOI:** 10.1001/jamanetworkopen.2024.24595

**Published:** 2024-08-13

**Authors:** Jun Chen, Jingjing Wang, Ziyi Qi, Shang Liu, Lingyi Zhao, Bo Zhang, Kaige Dong, Linlin Du, Jinliuxing Yang, Haidong Zou, Xiangui He, Xun Xu

**Affiliations:** 1 Shanghai Eye Diseases Prevention & Treatment Center, Shanghai Eye Hospital, School of Medicine, Tongji University, Shanghai, China.; 2 Department of Ophthalmology, Shanghai General Hospital, Shanghai Jiao Tong University, National Clinical Research Center for Eye Diseases, Shanghai, China

## Abstract

**Importance:**

Time spent outdoors has been proven effective in preventing myopia, but little is known about the association of outdoor exposure patterns with myopia.

**Objective:**

To examine the association of outdoor exposure patterns with myopic shift in children.

**Design, Setting, and Participants:**

This 1-year prospective cohort study from December 2017 to December 2018 was a secondary analysis of a cluster-randomized trial (Shanghai Time Outside to Reduce Myopia [STORM]). STORM was a school-based intervention study, recruiting 16 schools from 8 districts in Shanghai, from October 2016 to December 2018. Children without myopia at baseline who consistently wore a smartwatch for a minimum of 6 hours daily, sustained for at least 90 days, and who had complete information were included. Data analysis was performed from December 2017 to December 2018.

**Exposures:**

The outdoor exposure pattern was defined as the episode of time outdoors and instant sunlight intensity over a continuous period.

**Main Outcomes and Measures:**

Myopic shift was defined as the absolute change in refraction between the initial spherical equivalence and the follow-up spherical equivalence.

**Results:**

This study included 2976 students (mean [SD] age, 7.2 [0.6] years; 1525 girls [51.2%]). The mean (SD) daily time outdoors was 90 (28) minutes, and the mean (SD) sunlight intensity was 2345 (486) lux. Of the 12 outdoor exposure patterns, the major outdoor exposure patterns were time outdoors with at least 15 minutes, accounting for 74.9% of minutes (33 677 584 of 45 016 800 minutes). Only patterns with at least 15 minutes accompanied with no less than 2000 lux were associated with less myopic shift in refraction (for ≥15 minutes and 2000 to 3999 lux, −0.007 diopter [D] [95% CI, −0.011 to −0.002 D]; for ≥15 minutes and ≥4000 lux, −0.006 D [95% CI, −0.010 to −0.002 D]). The isotemporal substitution of patterns with at least 15 minutes and 2000 lux for other outdoor exposure patterns was positively associated with less myopic shift.

**Conclusions and Relevance:**

In this 1-year prospective cohort study of children with smartwatches, continuous outdoor exposure with at least 15 minutes accompanied with no less than 2000 lux sunlight intensity was associated with less myopic shift. These findings suggest that future outdoor interventions should focus not only on the overall time outdoors but also on the effective outdoor exposure patterns, as a means to effectively prevent myopia in children.

## Key Points

**Question:**

Are specific outdoor exposure patterns associated with the development of myopia in children?

**Findings:**

This prospective cohort study involving 2976 children equipped with smartwatches found that outdoor exposure patterns characterized by a continuous period of at least 15 minutes, accompanied by a sunlight intensity of more than 2000 lux, were associated with less myopic shift.

**Meaning:**

These findings suggest that future outdoor interventions for myopia prevention should include recommendations of outdoor exposure patterns of a minimum duration of 15 minutes and 2000 lux sunlight exposure.

## Teaser

This cohort study uses smartwatch data to determine whether outdoor exposure patterns are associated with myopic shift in children in Shanghai, China.

## Introduction

Myopia has emerged as a major global public health concern, with a sharp increase in cases over the past 3 decades.^1^ In East and Southeast Asia, the prevalence of myopia among young adults with 12 to 13 years of schooling has increased to nearly 90%.^2^ The trend of earlier onset of myopia among school-aged children suggests an increasingly serious epidemic of high myopia and pathologic myopia in the future.^3^ Higher degrees of myopia can increase the risk of vision-threatening conditions, such as myopic maculopathy, cataracts, and glaucoma, which not only impact individuals’ quality of life but also impose a substantial socioeconomic burden.^4,5^ Therefore, implementing effective interventions during early childhood is crucial for preventing and managing myopia.

Previous studies^6,7,8,9^ have demonstrated that spending more time outdoors is an effective intervention for myopia. The findings from randomized clinical trials^6,7,8^ revealed that children who engaged in increased outdoor activities exhibited a significantly lower incidence of myopia and experienced less myopic shift. Moreover, a meta-analysis^9^ concluded that extended time outdoors may reduce the onset of myopia. Animal studies^10,11,12^ involving chicks and rhesus monkeys have established that the intensity, duration, and frequency of light exposure impacted myopic shift. Human studies conducted by Read et al^13^ suggested that greater daily outdoor exposure (>3000 lux and >40 minutes per day) is likely to slow down axial elongation, whereas Wu et al^7^ claimed that spending shorter durations in brighter light or longer durations in moderate sunlight intensity (≥1000 or ≥3000 lux) can both provide protection against myopia. Recently, a myopia intervention study^14^ reported a dose-response association between outdoor light exposure and myopia incidence. The previous findings emphasized the impact of daily time outdoors and sunlight intensity on myopia, indicating a potential collaborative association between time outdoors and sunlight intensity in preventing the onset of myopia. Those studies^6,7,8,9^ recommended a minimum of 2 hours of time outdoors per day for this purpose.

However, there remain uncertainties surrounding the association between outdoor exposure pattern, particularly the impact of continuous outdoor exposure within a specific time frame, and myopic shift. Our study aims to investigate the association between outdoor exposure patterns and myopic shift among school-aged children participating in the Shanghai Time Outside to Reduce Myopia (STORM) study. It may contribute to optimizing the current outdoor intervention strategies among school-aged children by identifying outdoor exposure patterns associated with less myopic shift.

## Methods

### Study Design and Participants

This was a 1-year prospective cohort study nested in the STORM study, a school-based, cluster-randomized clinical trial conducted from October 2016 to December 2018 in Shanghai, China. Details of the methods have been reported previously.^15^ In the second year of the STORM study, from March 1 to December 31, 2018, each child was provided with a smartwatch to wear daily from 7:00 am to 7:00 pm.

Students wearing smartwatches were assessed for eligibility. Participants enrolling in the final analyses needed to meet the following criteria: (1) wearing the smartwatch for at least 6 hours per day; (2) wearing the smartwatch for at least 90 days during the study period; (3) not having myopia as of March 1, 2018; (4) having complete information on variables, including age, sex, spherical refraction equivalence, and axial length (AL); and (5) having no logic errors in the data.

The study was approved by the Shanghai General Hospital Ethics Committee and conformed with the tenets of the Declaration of Helsinki.^16^ Written informed consent for each child was obtained from a parent and/or caregiver. This study adhered to the Strengthening the Reporting of Observational Studies in Epidemiology (STROBE) reporting guidelines for cohort studies.

### Procedures

During the study, comprehensive eye examinations were conducted at the school by experienced physicians who had undergone standard training. Cycloplegia was performed using 1 drop of 0.5% proparacaine hydrochloride for topical anesthesia and 2 drops of 1% cyclopentolate (Cyclogyl; Alcon) 5 minutes apart. Forty minutes after instillation, pupillary size and response to light were assessed, and cycloplegia was considered complete for pupils larger than 6 mm with no response to light. In cases of incomplete dilation, an additional (third) cycloplegic drop was administered. Autorefraction using an automated refractor (KR-8900; Topcon) was then performed to measure the spherical equivalence, with 3 measurements taken and averaged. The AL was measured with an optical biometer (IOLMaster 500; Zeiss), with 5 measurements taken and averaged. Near-work time was investigated by questionnaires at each follow-up visit, including reading, taking indoor classes, and watching smartphones, pads, computers, and other electronic screens. Other covariates, including age, sex, parental myopia status, and body mass index (calculated as weight in kilograms divided by height in meters squared), were collected with questionnaires and anthropometric examinations.

Time outdoors and sunlight intensity were all objectively measured by the smartwatch. The wearable wristwatch was equipped with a light sensor, a global positioning system receiver module, and a pedometer. The light sensor sampled luminance (lux) and UV intensity at 20-second intervals. It sampled data once a minute. One piece of data consisted of time (year, month, day, hour, minute, and second), 3 data points on luminance, 3 data points on UV intensity, count of steps, weather, and wearing status. The data were automatically uploaded to the cloud disk to save the data. To distinguish between outdoor and indoor environments, a machine-learning algorithm was used, achieving a high accuracy of 92.4%, as reported in detail elsewhere.^17^

### Outcomes and Exposures

Myopic shift in refraction was defined as the absolute change in spherical equivalence between the initial spherical equivalence when participants wore the smartwatch and the follow-up spherical equivalence 1 year later. The outdoor exposure pattern was defined as the episode of time outdoors and instant sunlight intensity over a continuous period. The joint distribution of time outdoors and sunlight intensity over a continuous period was carefully considered in conjunction with practical interpretation for the categorization of outdoor exposure patterns. A total of 12 patterns were analyzed, as follows: pattern 1 (≤4 minutes and ≤1999 lux), pattern 2 (≤4 minutes and 2000-3999 lux), pattern 3 (≤4 minutes and ≥4000 lux), pattern 4 (5-9 minutes and ≤1999 lux), pattern 5 (5-9 minutes and 2000-3999 lux), pattern 6 (5-9 minutes and ≥4000 lux), pattern 7 (10-14 minutes and ≤1999 lux), pattern 8 (10-14 minutes and 2000-3999 lux), pattern 9 (10-14 minutes and ≥4000 lux), pattern 10 (≥15 minutes and ≤1999 lux), pattern 11 (≥15 minutes and 2000-3999 lux), and pattern 12 (≥15 minutes and ≥4000 lux).

The average sunlight intensity at the individual level was described with geometric means owing to the skewed distribution within the study period. Daily time outdoors at the individual level was computed by adding all the minutes and was standardized by the total days wearing the smartwatch.

### Statistical Analysis

Data analysis was performed from December 2017 to December 2018. Continuous variables with normal distributions, such as age, height, weight, baseline spherical equivalence, baseline AL, daily time outdoors, and average sunlight intensity, are presented as means and SDs. On the other hand, variables like near-work time, time outdoors with a continuous period, and sunlight intensity within a continuous period, which are characterized by a skewed distribution, are presented as medians and IQRs.

To assess the association between daily time outdoors and average sunlight intensity and myopic shift in refraction, multivariable linear regression was applied by adjusting age, sex, near-work time, parental myopia, and baseline spherical equivalence. The joint effect, main effect, and additive effect estimates between daily time outdoors and average sunlight intensity were estimated according to the following equation by VanderWeele^18^: *RERI* = *RD_T1L1_* − *RD_T1L0_* − *RD_T0L1_* + 1, where *T1* and *T0* indicate different levels of time outdoors, *L1* and *L0 *indicate different levels of light intensity, *RD* denotes risk difference, and *RERI* indicates relative excess risk due to interaction, which was the additive effect estimate in the current study. *RD_T1L1_* was the joint effect estimate. The proportions were attributed to time outdoors alone as *RD_T1L0 _/ RD_T1L1_*, light intensity alone as *RD_T0L1 _/ RD_T1L1_*, and the interaction as *RERI / RD_T1L1_*.

Regarding the potential presence of both interactions and nonlinearity at the same time between daily time outdoors and average sunlight intensity on spherical equivalence change, we furtherly applied restricted cubic spline (RCS) with an interaction term based on generalized linear regression to assess the association. For the sake of interpretation consistency, sunlight intensity was centralized to 2500 lux and rescaled to 1000 lux per unit, and time outdoors was centralized to 90 minutes per day and rescaled to 30 minutes per day per unit. To avoid overfitting, 3 knots were applied in RCS analysis on the basis of the lowest value of the Akaike Information Criterion. A contour plot was applied to display the interaction and nonlinear relationship between daily time outdoors and average sunlight intensity on spherical equivalence change.

The association between outdoor exposure patterns and myopic shift was assessed with residual method in the multivariable density model by adjusting cumulative time outdoors and cumulative sunlight intensity,^19^ as well as other covariates, including age, sex, near-work time, parental myopia, and baseline spherical equivalence. Standardized coefficients were computed to compare the relative importance of different outdoor exposure patterns. An isotemporal substitution model was applied to evaluate the time-substitution effect estimate of one outdoor exposure pattern to another while keeping cumulative time outdoors and sunlight intensity constant.^19^ Only patterns accounting for more than 5% of total minutes or total frequencies were included for evaluating their association with myopic shift. Sensitivity analysis was conducted to evaluate the stability by recategorizing outdoor exposure patterns, assessing the association of AL change and AL-to–corneal radius ratio change.

Estimated associations and 95% CIs were calculated, and statistical significance was set at 2-tailed *P* < .05. SAS statistical software version 9.4 (SAS Institute) and R statistical software version 4.2.2 (R Project for Statistical Computing) were used for data management and statistical analysis.

## Results

A total of 2976 children (mean [SD] age, 7.2 [0.6] years; 1525 girls [51.2%]; 1451 boys [48.8%]) with complete information were included for analysis (Table 1 and eFigure 1 in Supplement 1). Nearly one-half of the children’s parents (1144 children [48.5%]) did not have myopia. The median (IQR) near-work time was 239 (186-302) minutes per day. At baseline, the mean (SD) spherical equivalence was 1.26 (0.67) diopter (D), and the mean (SD) AL was 22.74 (0.69) mm.

**Table 1.** Characteristics of Study Participants

Variable	Participants (N = 2976)
Age, mean (SD), y	7.2 (0.6)
Sex, No. (%)	
Male	1451 (48.8)
Female	1525 (51.2)
Height, mean (SD), cm	125.5 (6.3)
Weight, mean (SD), kg	26.5 (5.6)
Body mass index, mean (SD)^a^	16.7 (2.6)
Parents with myopia, No. (%)	
Both	494 (16.6)
None	1144 (48.5)
Either	1038 (34.9)
Near-work time, median (IQR), min/d	239 (186-302)
Spherical equivalent refraction at baseline, mean (SD), diopter	1.26 (0.67)
Axial length at baseline, mean (SD), mm	22.74 (0.69)
Daily time outdoors, mean (SD), min/d	90 (28)
Sunlight intensity, mean (SD), lux	2345 (486)
Sunlight intensity within a continuous period, median (IQR), lux	2061 (1060-4812)
Time outdoors within a continuous period, median (IQR), min	11 (5-23)

^a^ Body mass index is calculated as weight in kilograms divided by height in meters squared.

eFigure 2 in Supplement 1 shows that the distribution of daily time outdoors and sunlight intensity was skewed to smaller values. Children tended to have more time outdoors on the weekdays, especially in the morning (8:00-9:00 am), midday (12:00-13:00 pm), and afternoon (15:00-16:00 pm). As Table 1 shows, the daily mean (SD) time outdoors was 90 (28) minutes per day, and the mean (SD) sunlight intensity was 2345 (486) lux. Within a continuous period of outdoor exposure, the median (IQR) sunlight intensity was 2061 (1060-4812) lux, and the median (IQR) time outdoors was 11 (5-23) minutes.

Table 2 and eFigure 3 in Supplement 1 depict a total of 12 different outdoor exposure patterns. Given the distribution of total frequencies, of the 12 outdoor exposure patterns, 491 878 exposures (17.2%) were pattern 1 and 530 345 exposures (18.6%) were pattern 12. Regarding the distribution of total minutes, the top 3 patterns were pattern 12 (16 251 597 minutes [36.1%]), pattern 10 (9 253 606 minutes [20.6%]), and pattern 11 (8 172 381 minutes [18.2%]), accounting for 74.9% of minutes (33 677 584 of 45 016 800 minutes).

**Table 2.** Distribution of Outdoor Exposure Patterns Among Participants During the Study Period

Outdoor exposure patterns^a^	Total frequency of exposures, No. (%)	Total minutes of exposure, No. (%)
Pattern 1 (≤4 min and ≤1999 lux)	491 878 (17.2)	1 391 811 (3.1)
Pattern 2 (≤4 min and 2000-3999 lux)	76 467 (2.7)	232 121 (0.5)
Pattern 3 (≤4 min and ≥4000 lux)	57 978 (2.0)	178 477 (0.4)
Pattern 4 (5-9 min and ≤1999 lux)	391 084 (13.7)	2 615 933 (5.8)
Pattern 5 (5-9 min and 2000-3999 lux)	138 493 (4.9)	964 427 (2.1)
Pattern 6 (5-9 min and ≥4000 lux)	134 418 (4.7)	951 349 (2.1)
Pattern 7 (10-14 min and ≤1999 lux)	186 521 (6.5)	2 189 012 (4.9)
Pattern 8 (10-14 min and 2000-3999 lux)	107 316 (3.8)	1 273 326 (2.8)
Pattern 9 (10-14 min and ≥4000 lux)	129 369 (4.5)	1 542 760 (3.4)
Pattern 10 (≥15 min and ≤1999 lux)	329 313 (11.5)	9 253 606 (20.6)
Pattern 11 (≥15 min and 2000-3999 lux)	284 230 (10.0)	8 172 381 (18.2)
Pattern 12 (≥15 min and ≥4000 lux)	530 345 (18.6)	16 251 597 (36.1)

^a^ The categorization of outdoor exposure patterns was carefully considered in conjunction with empirical scenarios and statistical distribution.

eTable 1 in Supplement 1 shows that the main effect estimate of daily time outdoors on absolute spherical equivalence change was −0.024 D (95% CI, −0.040 to −0.009 D; *P* = .002), and the main effect estimate of sunlight intensity on absolute spherical equivalence change was −0.026 D (95% CI, −0.058 to 0.005 D; *P* = .12). There was additive interaction between sunlight intensity and daily time outdoors on myopic shift (interaction effect estimate, −0.065 D; 95% CI, −0.098 to −0.032 D; *P* < .001); 56.2% (95% CI, 38.1% to 74.2%; *P* < .001) of the joint effect estimate can be attributed to the additive interaction. The RCS analysis showed that the daily time outdoors and sunlight intensity are both linearly associated with myopic shift (Figure, panels A and B). The contour plot (Figure, panel C) shows an unequal space between the contour lines, indicating that there was an additive interaction between sunlight intensity and daily time outdoors on myopic shift (*P* for interaction, <.001).

**Figure.**
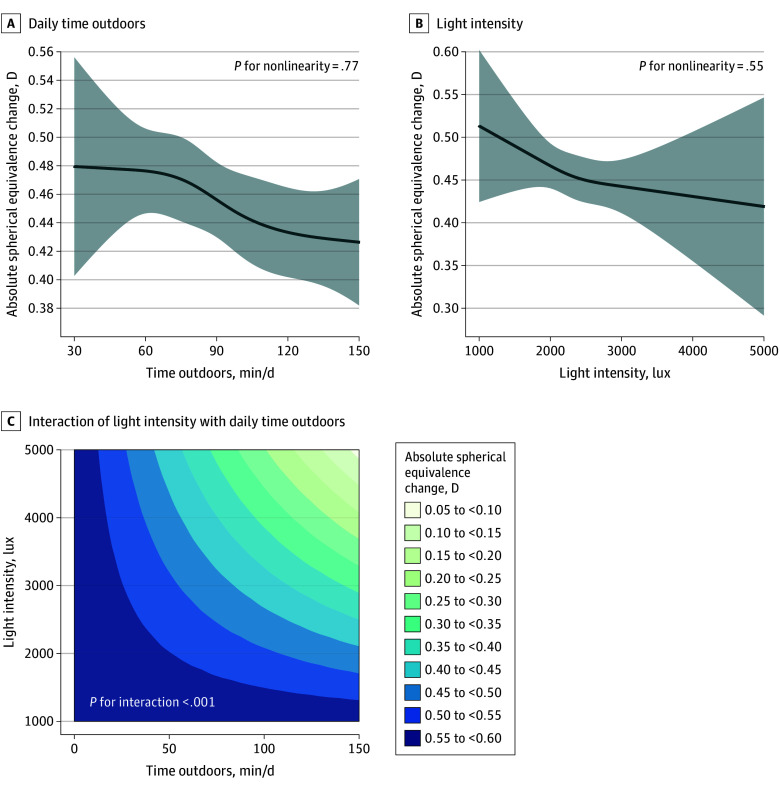
Association of Daily Time Outdoors and Light Intensity With Myopic Shift in Refraction In panels A and B, lines denote means, and shaded areas denote 95% CIs. D indicates diopter.

Table 3 shows that pattern 11 (−0.007 D; 95% CI, −0.011 to −0.002 D) and pattern 12 (−0.006 D; 95% CI, −0.010 to −0.002 D) are significantly associated with less myopic shift in refraction. The standardized coefficients indicated that pattern 12 had the larger association with less myopic shift compared with pattern 11. For example, for the absolute spherical equivalence change, the standardized estimate was −0.118 (95% CI, −0.207 to −0.029) for pattern 12 and −0.063 (95% CI, −0.110 to −0.015) for pattern 11. The trend analysis among pattern 10, pattern 11, and pattern 12 showed an association with increasing light intensity within a continuous period of more than 15 minutes (*P* for trend, .02).

**Table 3.** Association of Outdoor Exposure Patterns With Myopic Shift in Refraction Among Study Participants

**Outdoor exposure patterns** ^a^	**Coefficient (95% CI)** ^b^	**Standardized coefficient (95% CI)**
Patttern1 (≤4 min and ≤1999 lux)	−0.018 (−0.040 to 0.004)	−0.036 (−0.078 to 0.007)
Pattern 4 (5-9 min and ≤1999 lux)	−0.008 (−0.022 to 0.007)	−0.028 (−0.082 to 0.026)
Pattern 7 (10-14 min and ≤1999 lux)	0.000 (−0.012 to 0.012)	0.000 (−0.033 to 0.033)
Pattern 10 (≥15 min and ≤1999 lux)	−0.002 (−0.005 to 0.002)	−0.033 (−0.107 to 0.040)
Pattern 11 (≥15 min and 2000-3999 lux)	−0.007 (−0.011 to −0.002)^c^	−0.063 (−0.110 to −0.015)^c^
Pattern 12 (≥15 min and ≥4000 lux)	−0.006 (−0.010 to −0.002)^c^	−0.118 (−0.207 to −0.029)^c^
*P* value for trend^d^	NA	.02

Abbreviation: NA, not applicable.

^a^ A total of 2976 children were involved in the analyses for each outdoor exposure pattern. Residual method was applied for different outdoor exposure patterns in the multivariable density model with adjustment for age, sex, near-work time, parental myopia, baseline spherical equivalent, cumulative time outdoors, and cumulative sunlight intensity.

^b^ Coefficients are regression coefficients, and 95% CIs are calculated from the multivariable density models.

^c^ *P* < .05.

^d^ Trend analysis was applied for the standardized estimates in pattern 10, pattern 11, and pattern 12.

Table 4 shows that, in the context of isotemporal substitution models, the isotemporal substitution of pattern 11 or pattern 12 for other outdoor exposure patterns, while keeping cumulative time outdoors and sunlight intensity constant, was associated with less myopic shift. For instance, substituting pattern 11 for pattern 10 was associated with a reduced absolute spherical equivalence change of −0.005 D (95% CI, −0.009 to −0.001 D). However, when mutually substituting pattern 11 and pattern 12, both characterized by more than 15 minutes outdoor exposure, the analysis did not demonstrate a statistically significant association with myopic shift.

**Table 4.** Results of Isotemporal Substitution Model for Outdoor Exposure Patterns on Myopic Shift

Outdoor exposure patterns	Isotemporal substitution for each pattern, coefficient (95% CI)^a^	
	Pattern 11	Pattern 12
Pattern 1 (≤4 min and ≤1999 lux)	−0.006 (−0.011 to −0.001)^b^	−0.005 (−0.009 to −0.001)^b^
Pattern 4 (5-9 min and ≤1999 lux)	−0.006 (−0.011 to −0.001)^b^	−0.005 (−0.009 to −0.001)^b^
Pattern 7 (10-14 min and ≤1999 lux)	−0.007 (−0.011 to −0.002)^b^	−0.006 (−0.010 to −0.002)^b^
Pattern 10 (≥15 min and ≤1999 lux)	−0.005 (−0.009 to −0.001)^b^	−0.004 (−0.006 to −0.002)^b^
Pattern 11 (≥15 min and 2000-3999 lux)	NA	−0.002 (−0.005 to 0.001)
Pattern 12 (≥15 min and ≥4000 lux)	−0.002 (−0.006 to 0.002)	NA

Abbreviation: NA, not applicable.

^a^ The isotemporal substitution density model was adjusted for age, sex, near-work time, parental myopia, baseline spherical equivalent, and total time outdoors. The coefficients (95% CIs) represent the consequence of substituting 1% of that pattern instead of the substitution pattern while holding other patterns constant. Coefficients are the regression coefficients, and 95% CIs are calculated from the multivariable isotemporal substitution density models.

^b^ *P* < .05.

In the sensitivity analysis, eTable 2 in Supplement 1 presents the results of different categories of outdoor exposure patterns to verify the stability of the analysis. Multiple cutoffs were considered and estimated by multivariable linear regressions, and the results showed that only a continuous time outdoors larger than 15 minutes was statistically significantly associated with spherical equivalence change (15-19 minutes, −0.0006 D; 95% CI, −0.0012 to −0.0001 D; ≥20 minutes, −0.0002 D; 95% CI, −0.0003 to −0.0001 D). eTable 3 and eTable 4 in Supplement 1 show that the associations between outdoor exposure patterns and AL change were not statistically significant (*P* for trend, .16). eTable 5 and eTable 6 in Supplement 1 show that outdoor exposure patterns with more than 15 minutes (sunlight intensity included 2000-3999 lux and ≥4000 lux) were negatively associated with AL-to–corneal radius ratio change, although the *P* value for trend was not significant (*P* for trend, .08).

## Discussion

This 1-year, prospective cohort study with objectively monitored outdoor exposure data provides valuable insights into the association between outdoor exposure and myopic shift in children. Daily time outdoors and sunlight intensity were not as high as expected among children aged 7 to 9 years in Shanghai, China. Most children spent their outdoor time under a moderate sunlight intensity of less than 5000 lux. Time outdoors and sunlight intensity had an additive interaction on myopic shift. Outdoor exposure patterns of at least 15 minutes were found to be protective against myopic shift, with the association increasing with sunlight intensity.

Children in our study spent less time outdoors but were exposed to similar light levels, relative to their counterparts overseas. In the current study, half of the time outdoors was less than 90 minutes per day, and most average light levels were less than 2500 lux. This observation of time outdoors contrasted with reports from similarly aged children in Australia and the UK who reported spending at least 2 to 3 hours outdoors per day, but the light levels were similar to that of 1072 among Australian children aged 10 to 15 years and 1627 lux among US children aged 5 to 10 years.^13,20^ Differences in educational demands (homework load and after-school culturing classes), lifestyle, and sociocultural factors may account for the lower time outdoors among Chinese children.

This study found that time outdoors and sunlight intensity had an additive interaction on myopic shift. The protective effect of time outdoors concurs with existing studies that have highlighted the importance of increasing daily time spent outdoors in preventing myopia. Specifically, Chinese schoolchildren in Guangzhou who received a daily 40-minute outdoor intervention had a lower myopia incidence (difference, −9.1%; 30.4% vs 39.5%) and a less myopic shift (0.17 D; 95% CI, 0.01-0.33 D) in 3 years.^8^ An additional 80-minute period outdoors per day effectively reduced myopia incidence (difference, −9.3%; 8.4% vs 17.7%) over a shorter period of 1 year in a Taiwanese trial.^7^ The current findings suggest that the protective effect of time outdoors on myopia prevention is associated with the increasing sunlight intensity once it is higher than 2000 lux. Such moderate light-level conditions are easily attainable in most outdoor environments during daylight hours, under various street lighting (6000-16 000 lux) or even tree shade (5556-7876 lux) conditions.^21^ Thus, a practical recommendation would be to encourage longer time outdoors as much as possible, even under moderate light intensities.

The current study highlights the importance of outdoor exposure patterns with a continuous period of at least 15 minutes being associated with less myopic shift. This finding was consistent with an earlier randomized clinical trial in Sujiatun,^22^ which reported that an additional 20 minutes of outdoor activity in the morning and afternoon reduced myopia incidence. Although animal studies have suggested that shorter duration and higher frequency of light exposure might effectively slow down myopic shift,^12,23^ the current study suggests that outdoor exposure patterns with short durations may have minimal effects on myopic shift in humans. One of the possible explanations for this discrepancy is that the near-work time among humans is much longer than that for other species. Another explanation is related to the oxygen saturation in choroid vessels. The blood density would decrease after intense near work and longer outdoor exposure with stronger light levels could facilitate the recovery of blood intensity in choroid vessels efficiently.^24^ These findings could have important implications for the design and implementation of school-based and community-based outdoor programs aimed at increasing the effectiveness of time spent outdoors in preventing myopia.

### Limitations

Although this study provides valuable insights, several limitations should be acknowledged. First, the findings may not be directly generalizable to other geographic areas, because the study was limited to Shanghai. Second, a 1-year longitudinal study may not be sufficient to capture the associations between other outdoor exposure patterns and myopic shift, and this may induce biased estimation of the cutoff values. Longer-term studies would provide more robust evidence. Third, the use of wrist smartwatches to estimate light levels may lead to underestimation compared with actual light levels reaching the eyes. Therefore, further research is needed to validate these findings in diverse populations and explore the potential benefits of outdoor interventions in different settings.

## Conclusions

In conclusion, continuous outdoor exposure of at least 15 minutes accompanied with no less than 2000 lux sunlight intensity was associated with less myopic shift. These findings suggest that future outdoor intervention programs should focus not only on daily time outdoors but also the outdoor exposure patterns to prevent myopia.

## Notes

Supplement 1. **eTable 1.** The joint effect, main effect and interaction between daily time outdoors and sunlight intensity on myopic shift in refraction **eTable 2.** Results of multivariable linear regression models for different cutoffs of outdoor exposure patterns **eTable 3.** The effect of outdoor exposure patterns on myopic shift in axial length change among participants in the study **eTable 4.** Results of isotemporal substitution model for outdoor exposure patterns on myopic shift in axial length change **eTable 5.** The effect of outdoor exposure patterns on myopic shift in ALCR change among participants in the study **eTable 6.** Results of isotemporal substitution model for outdoor exposure patterns on myopic shift in ALCR change **eFigure 1.** Flowchart of the assessment process for participants in the prospective study **eFigure 2.** Distribution of daily time outdoors and sunlight intensity during the study period **eFigure 3.** The distribution the twelve outdoor exposure patterns among participants during the study period

Supplement 2.
Data Sharing Statement
