# Supplementary material for: Smartwatch Measures of Outdoor Exposure and Myopia in Children
Source: JAMA Netw Open. 2024 Aug 13;7(8):e2424595. doi: 10.1001/jamanetworkopen.2024.24595 (PMC11322842; doi:10.1001/jamanetworkopen.2024.24595)
Supplement: Supplement 1. — eTable 1. The joint effect, main effect and interaction between daily time outdoors and sunlight intensity on myopic shift in refraction eTable 2. Results of multivariable linear regression models for different cutoffs of outdoor exposure patterns eTable 3. The effect of outdoor exposure patterns on myopic shift in axial length change among participants in the study eTable 4. Results of isotemporal substitution model for outdoor exposure patterns on myopic shift in axial length change eTable 5. The effect of outdoor exposure patterns on myopic shift in ALCR change among participants in the study eTable 6. Results of isotemporal substitution model for outdoor exposure patterns on myopic shift in ALCR change eFigure 1. Flowchart of the assessment process for participants in the prospective study eFigure 2. Distribution of daily time outdoors and sunlight intensity during the study period eFigure 3. The distribution the twelve outdoor exposure patterns among participants during the study period [file jamanetwopen-e2424595-s001.pdf]

Supplemental Online Content

Chen J, Wang J, Qi Z, et al. Smartwatch measures of outdoor exposure and myopia in children. *JAMA Netw Open*. 2024;7(8):e2424595. doi:10.1001/jamanetworkopen.2024.24595

eTable 1. The joint effect, main effect and interaction between daily time outdoors and sunlight intensity on myopic shift in refraction
eTable 2. Results of multivariable linear regression models for different cutoffs of outdoor exposure patterns
eTable 3. The effect of outdoor exposure patterns on myopic shift in axial length change among participants in the study
eTable 4. Results of isotemporal substitution model for outdoor exposure patterns on myopic shift in axial length change
eTable 5. The effect of outdoor exposure patterns on myopic shift in ALCR change among participants in the study
eTable 6. Results of isotemporal substitution model for outdoor exposure patterns on myopic shift in ALCR change
eFigure 1. Flowchart of the assessment process for participants in the prospective study
eFigure 2. Distribution of daily time outdoors and sunlight intensity during the study period
eFigure 3. The distribution the twelve outdoor exposure patterns among participants during the study period

This supplemental material has been provided by the authors to give readers additional information about their work.

© 2024 Chen J et al. *JAMA Network Open.*

eTable 1 The joint effect, main effect and interaction between daily time outdoors and sunlight intensity on myopic shift in refraction

	**Absolute SE Change** [r]	***P* value**
**Joint Effects**[†]	-0.116 (-0.164~-0.069)	<0.001
**Main Effects**[#]		
Average light intensity (1000 lux per unit)	-0.026 (-0.058~0.005)	0.116
Daily time outdoors (30 mins/day per unit)	-0.024 (-0.040~-0.009)	0.002
**Effects due to interaction**		
Additive interaction	-0.065 (-0.098~-0.032)	<0.001
**Attributable Proportion, (%)**		
Average light intensity (1000 lux per unit)	22.5 (0.6, 45.0)	0.057
Daily time outdoors (30 mins/day per unit)	21.3 (7.7, 34.9)	0.002
Additive interaction	56.2 (38.1, 74.2)	<0.001

Abbreviations: SE for spherical equivalent.

† Joint effect referred to the combined effect from time outdoors and sunlight intensity on myopic shift.

# Sunlight intensity was centralized to 2500lux and rescaled to 1000 lux per unit and time outdoors was centralized to 90mins/day and rescaled to 30 mins/day per unit.

r Generalized linear regression model was applied by adjusting age, gender, near-wok time, parental myopia and baseline spherical equivalence.

© 2024 Chen J et al. *JAMA Network Open.*

eTable 2 Results of multivariable linear regression models for different cutoffs of outdoor exposure patterns

Different categories of outdoor exposure patterns	Estimate (95%*CI*) #
≤4mins	-0.0002 (-0.0005, 0.0002)
5-9mins	-0.0001 (-0.0004, 0.0002)
10~14mins	-0.0001 (-0.0006, 0.0003)
15-19mins	-0.0006 (-0.0012, -0.0001)*
≥20mins	-0.0002 (-0.0003, -0.0001)*

# Generalized linear regression model with adjusting age, gender, near-wok time, parental myopia, baseline spherical equivalent, cumulative time outdoors and cumulative sunlight intensity.

* *P* value less than 0.05.

© 2024 Chen J et al. *JAMA Network Open.*

eTable 3 The effect of outdoor exposure patterns on myopic shift in axial length change among participants in the study

**Outdoor exposure patterns** [r]	**Coefficients (95%*CI*)**	**Standardized Coefficients**
Patttern1 (≤4mins and ≤1999lux)	−0. 002 (−0. 011, 0. 007)	−0. 008 (−0. 051, 0. 034)
Pattern4 (5~9mins and ≤1999lux)	−0. 003 (−0. 009, 0. 003)	−0. 027 (−0. 081, 0. 027)
Pattern7 (10~14mins and ≤1999lux)	0. 002 (−0. 003, 0. 007)	0. 019 (−0. 026, 0. 064)
Pattern10 (≥15mins and ≤1999lux)	0. 001 (−0. 001, 0. 002)	0. 04 (−0. 035, 0. 115)
Pattern11 (≥15mins and 2000~3999lux)	−0. 001 (−0. 003, 0. 001)	−0. 018 (−0. 062, 0. 029)
Pattern12 (≥15mins and ≥4000lux)	−0. 001 (−0. 003, 0. 001)	−0. 057 (−0. 149, 0. 031)
P for trend [Ł]	-	0. 163

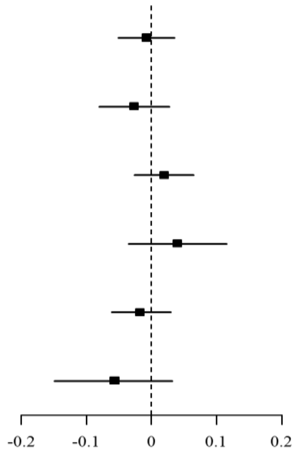

Coefficients (95%*CI*) were the regression coefficients and 95% confidence interval from the multivariable density models.

r Residual method was applied for different outdoor exposure patterns in the multivariable density model with adjusting age, gender, near-wok time, parental myopia, baseline spherical equivalent, cumulative time outdoors and cumulative sunlight intensity.

***** *P*-value less than 0.05.

Ł Tend analysis was applied for the standardized estimates in pattern 10, pattern 11 and pattern 12.

© 2024 Chen J et al. *JAMA Network Open.*

eTable 4 Results of isotemporal substitution model for outdoor exposure patterns on myopic shift in axial length change

**Outdoor exposure patterns**	**Isotemporal substitution for each pattern** [r]	
	Pattern 11<br>Coefficients (95%*CI*)	Pattern 12<br>Coefficients (95%*CI*)
Patttern1 (≤4mins and ≤1999lux)	-0.001(-0.003,0.001)	-0.001(-0.003,0.001)
Pattern4 (5~9mins and ≤1999lux)	-0.001(-0.002,0.002)	-0.001(-0.002,0.001)
Pattern7 (10~14mins and ≤1999lux)	-0.001(-0.003,0.001)	-0.001(-0.003,0.001)
Pattern10 (≥15mins and ≤1999lux)	-0.001(-0.003,0.000)	-0.002(-0.003,-0.001)*
Pattern11 (≥15mins and 2000~3999lux)	——	-0.001(-0.002,0.001)
Pattern12 (≥15mins and ≥4000lux)	0.000(-0.001,0.002)	——

Coefficients (95%*CI*) were the regression coefficients and 95% confidence interval from the multivariable isotemporal substitution density models.
r Isotemporal substitution density model was adjusted for age, gender, near-wok time, parental myopia, baseline spherical equivalent and total time outdoors. The coefficients (95%CI) represent the consequence of substituting one percent of that pattern instead of the substitution pattern while holding other patterns constant.
***** *P*-value less than 0.05.

© 2024 Chen J et al. *JAMA Network Open.*

eTable 5 The effect of outdoor exposure patterns on myopic shift in ALCR change among participants in the study

**Outdoor exposure patterns** [r]	**Coefficients (95%*CI*)**	**Standardized Coefficients**
Patttern1 (≤4mins and ≤1999lux)	−0. 0005 (−0. 0018, 0. 0009)	−0. 015 (−0. 053, 0. 027)
Pattern4 (5~9mins and ≤1999lux)	−0. 0006 (−0. 0014, 0. 0003)	−0. 034 (−0. 08, 0. 017)
Pattern7 (10~14mins and ≤1999lux)	0. 0000 (−0. 0007, 0. 0007)	0. 001 (−0. 009, 0. 011)
Pattern10 (≥15mins and ≤1999lux)	0. 0000 (−0. 0002, 0. 0002)	0. 011 (−0. 022, 0. 033)
Pattern11 (≥15mins and 2000~3999lux)	−0. 0003 (−0. 0006, −0. 0000)	−0. 048 (−0. 095, 0. 000)
Pattern12 (≥15mins and ≥4000lux)	−0. 0003 (−0. 006, −0. 0001)	−0. 112 (−0. 225, −0. 037)
P for trend [Ł]	-	0. 076

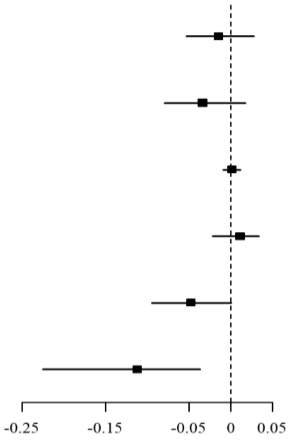

ALCR: the ratio of axial length to corneal curvature radius.

Coefficients (95%*CI*) were the regression coefficients and 95% confidence interval from the multivariable density models.

r Residual method was applied for different outdoor exposure patterns in the multivariable density model with adjusting age, gender, near-wok time, parental myopia, baseline spherical equivalent, cumulative time outdoors and cumulative sunlight intensity.

***** *P*-value less than 0.05.

Ł Tend analysis was applied for the standardized estimates in pattern 10, pattern 11 and pattern 12.

© 2024 Chen J et al. *JAMA Network Open.*

eTable 6 Results of isotemporal substitution model for outdoor exposure patterns on myopic shift in ALCR change

Outdoor exposure patterns	Isotemporal substitution for each pattern [r]	
	Pattern 11<br>Coefficients (95%*CI*)	Pattern 12<br>Coefficients (95%*CI*)
Patttern1 (≤4mins and ≤1999lux)	-0.0003(-0.0006,0.0000)	-0.0003(-0.0006,-0.0001)*
Pattern4 (5~9mins and ≤1999lux)	-0.0002(-0.0005,-0.0001)*	-0.0003(-0.0005,-0.0001)*
Pattern7 (10~14mins and ≤1999lux)	-0.0003(-0.0006,0.0000)*	-0.0003(-0.0006,-0.0001)*
Pattern10 (≥15mins and ≤1999lux)	-0.0003(-0.0006,-0.0001)*	-0.0004(-0.0005,-0.0002)*
Pattern11 (≥15mins and 2000~3999lux)	——	-0.0002(-0.0003,0.0001)
Pattern12 (≥15mins and ≥4000lux)	0.0000(-0.0003,0.0003)	——

ALCR: the ratio of axial length to corneal curvature radius.

Coefficients (95%*CI*) were the regression coefficients and 95% confidence interval from the multivariable isotemporal substitution density models.

r Isotemporal substitution density model was adjusted for age, gender, near-wok time, parental myopia, baseline spherical equivalent and total time outdoors. The coefficients (95%CI) represent the consequence of substituting one percent of that pattern instead of the substitution pattern while holding other patterns constant.

***** *P*-value less than 0.05.

© 2024 Chen J et al. *JAMA Network Open.*

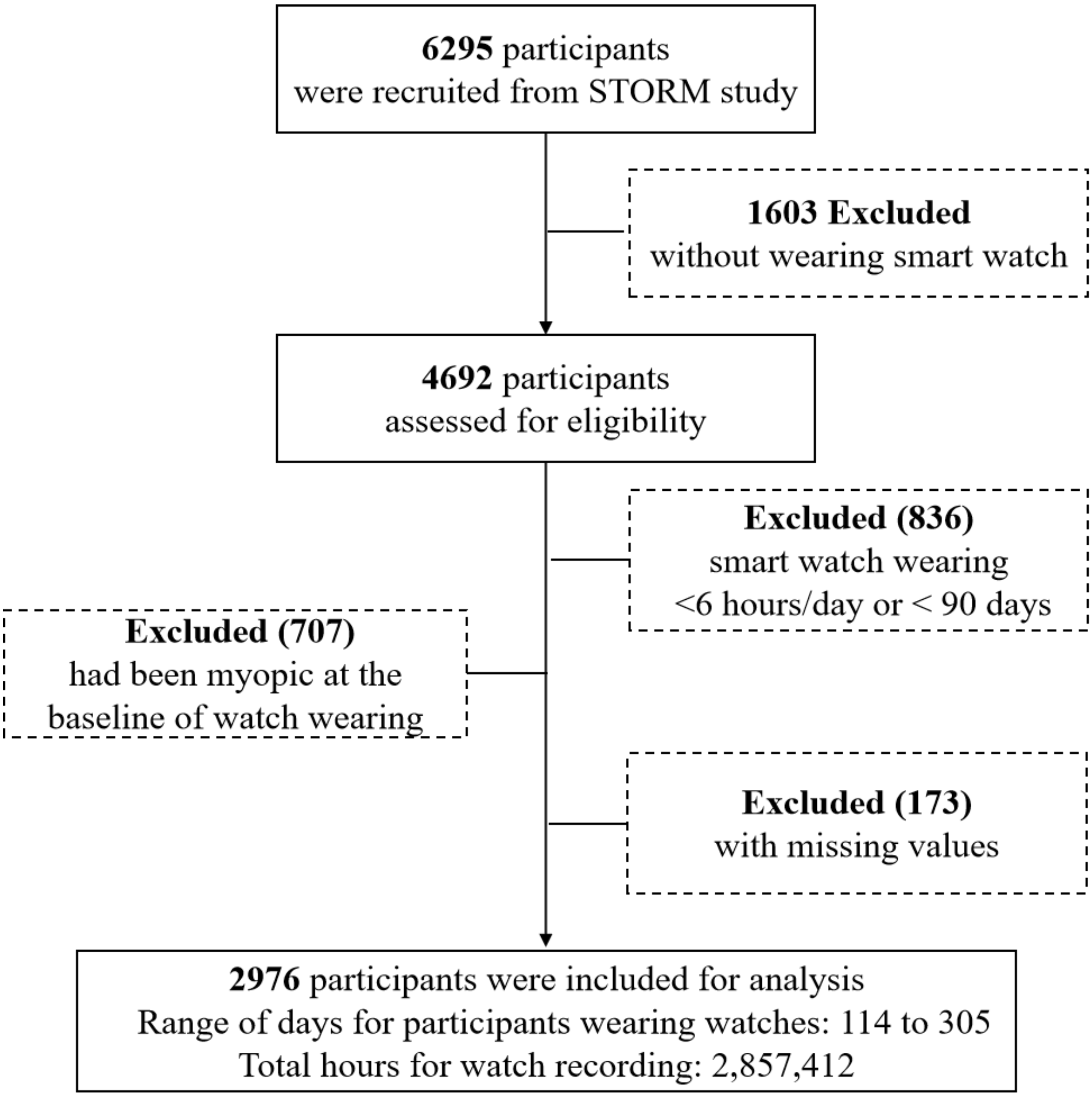

eFigure 1 Flowchart of the assessment process for participants in the prospective study.

© 2024 Chen J et al. *JAMA Network Open.*

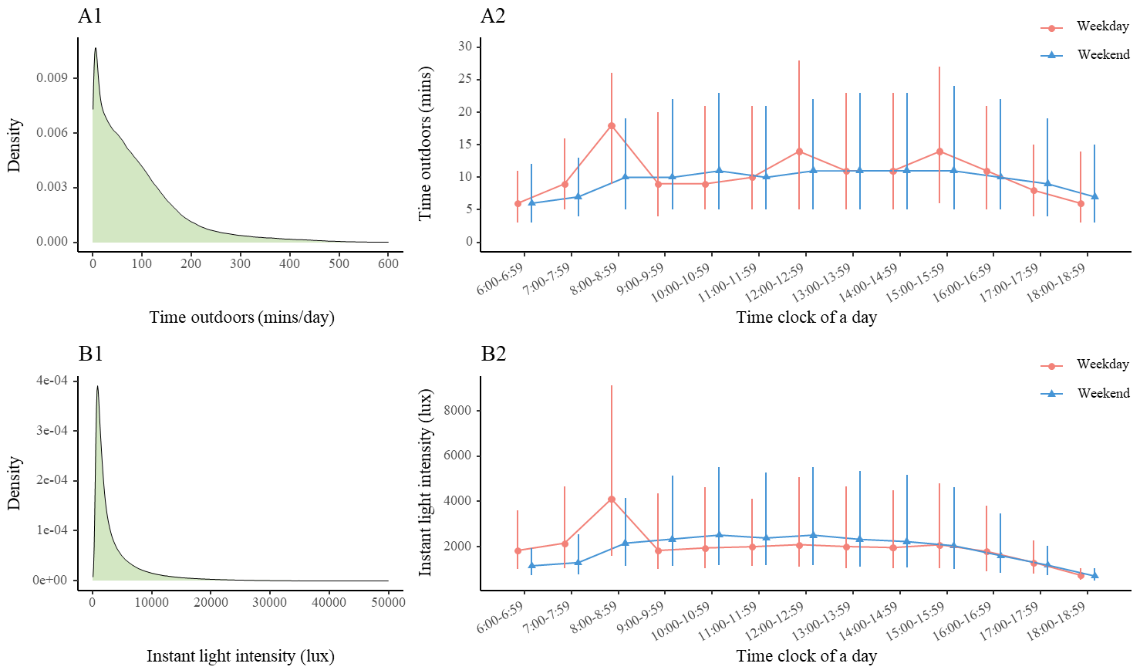

eFigure 2 Distribution of daily time outdoors and sunlight intensity during the study period. (A1) is for the distribution of daily time outdoors among all participants during the study period. (A2) is to describe the minutes of time outdoors per hour for all participants from 6:00 to 19:00. The red dotted line indicated the weekdays and the blue dotted line indicated the weekends. The dotted points and error bars indicated the median and quartiles. (B1) is for the distribution of sunlight intensity among all participants during the study period. (B2) is to describe the sunlight intensity per hour for all participants from 6:00 to 19:00. The red dotted line indicated the weekdays and the blue dotted line indicated the weekends. The dotted points and error bars indicated the median and quartiles.

© 2024 Chen J et al. *JAMA Network Open.*

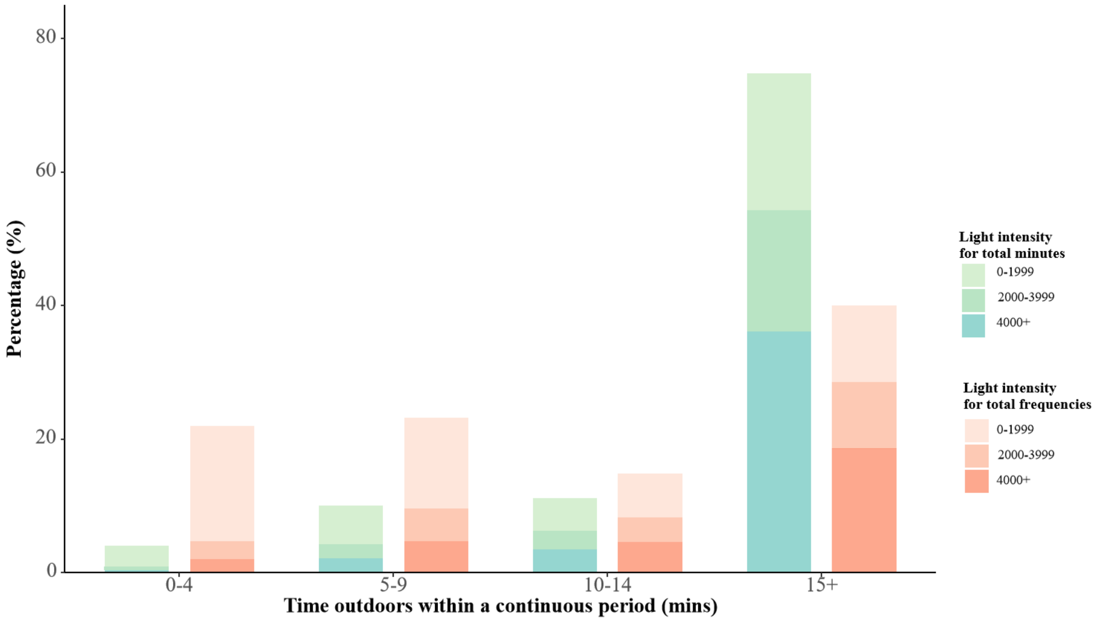

eFigure 3 The distribution the twelve outdoor exposure patterns among participants during the study period. The green block indicated the percentage of minutes of each outdoor pattern to total minutes of outdoor exposure patterns. The orange block indicated the percentage of frequencies of each outdoor pattern to total frequencies of outdoor exposure patterns.

© 2024 Chen J et al. *JAMA Network Open.*
