# Supplementary material for: Smartwatch Measures of Outdoor Exposure and Myopia in Children
Source: JAMA Netw Open. 2024 Aug 13;7(8):e2424595. doi: 10.1001/jamanetworkopen.2024.24595 (PMC11322842; doi:10.1001/jamanetworkopen.2024.24595)
Supplement: Supplement 2. — Data Sharing Statement [file jamanetwopen-e2424595-s002.pdf]

Data Sharing Statement

Chen. Smartwatch Measures of Outdoor Exposure and Myopia in Children. *JAMA Netw Open*. Published August 13, 2024. doi:10.1001/jamanetworkopen.2024.24595

Data
Data available: No
